# A Case of Complicated Survivorship Care

**Published:** 2014-01-01

**Authors:** Jean Rosiak, Candi Humphreys

**Affiliations:** From Aurora Health Care/Aurora Sinai Comprehensive Breast Care Center, Milwaukee, Wisconsin; Concordia University Wisconsin, Mequon, Wisconsin

## Abstract

At age 34, Ms. P., a premenopausal African American woman, was diagnosed with stage IA poorly differentiated invasive ductal carcinoma of the left breast. Her tumor was estrogen and progesterone receptor (PR) positive but HER2 negative. Ms. P.’s treatment included a partial mastectomy, radiation therapy, and 2 years of tamoxifen, although tamoxifen was discontinued due to pregnancy. She was then lost to follow-up.

At age 40, Ms. P. discovered a mass in her left breast upon self- exam. There was a delay in diagnosis due to pregnancy, which was terminated. A core biopsy was performed, and the pathology was positive for poorly differentiated invasive ductal carcinoma that was ER and PR positive but HER2 negative. She was clinically stage IIIB, with the tumor fixed to the chest wall.

Ms. P.’s family history included premenopausal breast cancer in her mother, three of her maternal aunts, and her maternal grandmother. Genetic testing was performed; she was found to have a BRCA2 deleterious mutation.

After an intrauterine device (IUD) was placed to prevent pregnancy, Ms. P. began neoadjuvant chemotherapy consisting of doxorubicin and cyclophosphamide for 4 cycles followed by paclitaxel for 4 cycles. She then underwent a left salvage mastectomy and sentinel lymph node biopsy. After treatment, she was assessed at pathologic stage IA. Tamoxifen was reinitiated. After completing treatment, Ms. P. presented for a survivorship visit accompanied by her fiancé.

In 2006, the Institute of Medicine (IOM) issued the report "From Cancer Patient to Cancer Survivor: Lost in Transition" (Hewitt, Greenfield, & Stovall, 2006), addressing the problem of lack of coordinated care for cancer survivors after completion of treatment. The IOM recommends that each patient completing treatment for their malignancy be provided with a survivorship care plan. The purpose of this plan is to summarize details of the diagnosis and treatment received, provide a schedule for future care, identify which providers will be involved in follow-up care, address lifestyle modifications that can decrease the risk of tumor recurrence and improve overall health, and inform the patient regarding psychosocial services available in the community. At any point in time, one or more of these areas of concern may take priority in the patient’s life.

In a study conducted by Ness et al. (2013), the top two concerns reported by cancer survivors, extending for years following treatment, are fear of recurrence and uncertainty. Providing information that clarifies follow-up, conveys information regarding long-term and late effects of treatment, and educates about signs of recurrence may lessen this fear and uncertainty (Curcio, Lambe, Schneider, & Khan, 2012; Kimman et al., 2011). At times, however, more immediate issues can override these common concerns.

## Survivorship Visit 1

A survivorship care plan that summarized her individual disease and treatment to date, outlined the recommended follow-up care, and suggested lifestyle changes for improving her health and decreasing the risk of recurrence was prepared for Ms. P. and presented to her and her fiancé. The recommended follow-up plan was based on American Society of Clinical Oncology (ASCO) guidelines (Khatcheressian et al., 2013).

During this visit, it was noted that Ms. P. had resumed smoking and had gained weight, with a body mass index (BMI) increase from 23 to 24.5 over a 6-month period. She seemed uninterested in discussing these issues. She stated that she was too busy providing continual care for her fiancé’s ailing mother to exercise or eat properly, and that she had resumed smoking due to stress.

An attempt was made to discuss Ms. P.’s BRCA2-positive status and the increased risk this posed for additional malignancy. An attempt was also made to determine whether she had informed her family members about this mutation and its significance for them. Ms. P. exhibited poor understanding of the implications of her positive genetic test for herself and her family members and reported that she had not shared the results. She was referred to the genetic counselor for a second discussion of the results and their relevance to herself and her family.

## Implications of Positive Genetic Testing

A BRCA2 mutation has implications for the individual as well as for her relatives. The National Comprehensive Cancer Network (NCCN, 2013) provides guidelines for the testing and follow-up of these individuals. A person identified as having a BRCA2 mutation has approximately a 49% to 55% risk of developing a contralateral breast cancer and a 16.5% to 18% risk of developing ovarian cancer (NCCN, 2013). Risk-reducing surgery should be discussed, including prophylactic mastectomy and/or salpingo-oophorectomy. A surveillance plan to monitor for second malignancies should be in place.

It is critical that the implications for family members be explained to the individual. Each first-degree relative has a 50% chance of having the same BRCA mutation as a carrier, and each second-degree relative has a 25% chance of sharing the mutation (Roesser, 2010). A discussion should be held with the patient regarding who should be informed and how this information should be disclosed (Greco & Goetsch, 2010). Permission to share this information should be documented.

The genetic counselor obtained Ms. P.’s permission to disclose the results to her siblings, her adult children, and the guardians of her 3 children under age 18. In this way, the correct information would be relayed and recommendations for risk reduction and surveillance would be conveyed.

## Survivorship Visit 2

Ms. P. presented 3 months later, accompanied by her fiancé; he waited for her in the waiting room and talked on his cell phone. She smelled heavily of smoke and had gained weight. She began crying, stating that she was in an abusive relationship. She feared she was suffering from broken ribs as a result of recent violence. Ms. P. admitted long-term physical and psychological abuse and stated that her fiancé threatened to kill her if she left. He said he needed her to continue taking care of his mother, but she no longer wanted that responsibility. He had isolated her from her family and friends, locking her in the house and taking her cell phone with him when he went to work so she could not contact anyone unless he was present. She also feared she was pregnant, and she stated that she was still on tamoxifen.

## Domestic Abuse

One in four women experiences domestic violence during her lifetime (American Bar Association [ABA], 2011; Black et al., 2011; National Coalition Against Domestic Violence [NCADV], 2007). Almost half of US women have experienced some sort of psychological aggression from an intimate partner (Black et al., 2011). Intimate partner violence (IPV)—including physical, sexual, emotional, and economic abuse—is most often accompanied by dominating and controlling behavior that includes attempting to isolate the victim from family and friends (Mick, 2006; NCADV, 2007). Victims of IPV are more likely to suffer from physical maladies and poor mental health (Black et al., 2011; Mick, 2006). In 2007, IPV accounted for 14% of all homicides, and approximately 33% of female homicides were committed by an intimate partner (ABA, 2011; Black et al., 2011; Catalano, Smith, Snyder, & Rand, 2009). Black women are more likely to experience IPV than White women, and they are four times more likely to be killed by an intimate partner (Catalano et al., 2009). Access to firearms increases the risk of intimate partner homicide fivefold (ABA, 2011).

Only one-third of women report domestic abuse to their health-care provider (Goroll & Mulley, 2009). Common themes that prevent the disclosure of IPV include fear for safety of self and/or others (i.e., children), hope that the situation may improve, shame or embarrassment, financial constraints, fear of retaliation, and concern that support from law enforcement may not be available (Black et al., 2011; Mick, 2006; Papadakis & McPhee, 2013).

Barriers to health-care providers identifying abuse include reluctance of the patient to bring up the issue, time limitations on visits, focus of the provider on other concerns during the visit, and uncertainty about how to respond effectively to the disclosure of abuse (Goroll & Mulley, 2009). Providers should be proactive in identifying women potentially experiencing abuse, either by regularly screening patients or approaching patients with chronic unexplained or nonspecific complaints regarding their personal situation. Indicating concern for the patient’s safety may facilitate their disclosure of IPV (Mick, 2006). It is critical to examine these individuals when unaccompanied by family or friends (Goroll & Mulley, 2009; Mick, 2006). Some states require that health-care providers report suspected domestic abuse (Papadakis & McPhee, 2013).

Interventions to assist patients in dealing with domestic abuse include providing counseling support; educating about legal rights; identifying interventions, resources, and a safe place; assisting with the development of an emergency escape plan; and maintaining confidentiality (Goroll & Mulley, 2009; Mick, 2006). The woman in question should be given contact information for local or institutional domestic abuse assistance. National resources include the National Domestic Violence Hotline at 1-800-799-SAFE (7233) and the National Sexual Assault Hotline at 1-800-656-4673 (NCADV, 2007).

Ms. P. was provided with contact information for the above hotlines. A call was placed to the institution’s hotline for domestic abuse, and the patient spoke with the counselor. A plan to leave the situation was developed. Ms. P.’s 21-year old daughter agreed to take her into her home, the location of which was unknown to Ms. P.’s fiancé. A plan to prepare her belongings and to accumulate as much money as she could in preparation for leaving the situation was developed. A time when she would be able to leave the situation was determined.

Ms. P. was sent for rib x-rays, which did not identify any fractures. Her pregnancy test came back negative. An ultrasound did not identify the previously placed IUD, although Ms. P. was not sure what had happened to it or how it had been removed. The importance of avoiding pregnancy while on tamoxifen was again stressed.

## Survivorship Visit 3

Ms. P. returned for another follow-up visit 3 months later. She had successfully left her fiancé and was living with her daughter. Her fiancé had initially tried to find her, but family members had convinced him to avoid further contact. She had stopped smoking and reported making better eating choices. She had continued on tamoxifen. Ms. P. stated that her daughter had asked her to find another place to live, but that she was unsure where to go; she was not working and her income was limited. She was considering moving south to be near family members who might take her in. Compliance with follow-up was a significant concern.

## Noncompliance in Cancer Care

Adherence to a medical plan for the management of health-related conditions is an issue in every health-care practice. Up to 50% of patients do not adhere to recommendations, and as many as one-third never take their medications (Miaskowski, Shockney, & Chlebowski, 2008; Papadakis & McPhee, 2013). When conditions are chronic and treatment is long term, is associated with adverse effects, or has intangible benefits, there is particular difficulty with compliance. History of noncompliance, lack of social support, and the presence of psychological problems such as depression are also predictors of poor adherence. By educating patients so that they believe in the importance of their prescribed medication, determining whether there are financial issues that prevent compliance, developing a therapeutic and supportive relationship with the patient, and connecting the patient with support groups and organizations to contact for more information, health-care professionals can help to increase adherence to the recommended treatment (Miaskowski, Shockney, & Chlebowski, 2008).

Ms P. had been lost to follow-up by her initial oncology providers. She had a history of noncompliance, and the risk for continuation of this trend was high. She was provided with another copy of her survivorship care plan and was encouraged to keep it as well as share it with any new providers she sees in the future. The importance of continuing tamoxifen to decrease her risk of recurrent disease was emphasized.

## Conclusion

The case of Ms. P. illustrates the complexity of providing survivorship care and emphasizes the consideration that must be given to the psychosocial problems patients may face while they deal with the impact of cancer. It remains to be seen whether Ms. P. will continue on her care path or once again be lost to appropriate follow-up during this life transition.
